# 4299 The University of North Carolina CTSA Hub (NC TraCS) Service Evaluation: Using Customer Feedback to Improve Services

**DOI:** 10.1017/cts.2020.244

**Published:** 2020-07-29

**Authors:** Shayne Thomas McKinley, Tanha Patel, Tim Carey, John B Buse, Andrea Carnegie, Giselle Corbie-Smith, Gaurav Dave

**Affiliations:** 1 University of North Carolina School of Medicine; 2 NC TraCS

## Abstract

OBJECTIVES/GOALS: The North Carolina Translational and Clinical Sciences Institute (NC TraCS) supports faculty and staff in carrying out clinical and translational research at UNC-Chapel Hill. To better understand customer satisfaction and impact, a survey was administered among NC TraCS users. METHODS/STUDY POPULATION: NC TraCS has 13 program areas that range from Biostatistics to Community and Stakeholder Engagement. These programs provide services to faculty, staff, students, and outside researchers in the area of clinical and translational science. A customer feedback survey was administered in Spring 2019 to anyone who had used at least one NC TraCS service between March 1^st^, 2017 and February 28^th^, 2019. A total of 856 survey invitations were sent. The survey included questions around users’ perception of the ease of access, helpfulness, outcome, and promptness of the services received using 6-point Likert scale. The survey also addressed career impact, communications, and suggestions for improvement. RESULTS/ANTICIPATED RESULTS: We received 268 responses, (31% response). Majority of respondents were satisfied with Overall Helpfulness (95%), Outcome of Service (96%), Ease of Access (93%), and Promptness of Service (90%). They also noted that their careers had at least slightly improved in the following areas: Mentorship (76%), Research Methods (75%), Skill Development (77%), Research Direction (71%) and Collaboration (80%). Furthermore, 96% responded positively to returning to TraCS. The feedback received was shared with service administrators and NC TraCS leadership to identify areas of improvement and further strengthen their services. Concerns, when present, were addressed by service directors or the overall PI’s. DISCUSSION/SIGNIFICANCE OF IMPACT: Need to communicate expectations to customers the expected turn-around time for help emerged as a clear take-away. In response, TraCS leadership is working to improve staffing and workflows for efficient service delivery including expectation management, especially among the most popular services.

